# A randomized clinical trial: timing of indwelling urethral catheter removal following transurethral resection of prostate

**DOI:** 10.2144/fsoa-2023-0195

**Published:** 2024-05-20

**Authors:** Saddam Al Demour, Mohammad T Al-Zubi, Mera Ababneh, Samer F Al-Rawashdah, Muayyad Ahmad

**Affiliations:** 1 Department of Special Surgery, Division of Urology, School of Medicine, The University of Jordan, Amman, 11942, Jordan; 2 Dr Sulaiman Al Habib Medical Group, Riyadh, 12214, Kingdome of Saudi Arabia; 3 Department of Urology, School of Medicine, Yarmouk University, Irbid, 21110, Jordan; 4 Department of Clinical Pharmacy, Faculty of Pharmacy, Jordan University of Science and Technology, Irbid, 22110, Jordan; 5 Department of Special Surgery, Urology Unit, School of Medicine, Mutah University, Karak, 61710, Jordan; 6 Clinical Nursing Department, School of Nursing, The University of Jordan, Amman, 11942, Jordan

**Keywords:** benign prostatic hyperplasia, early versus delay catheter removal, transurethral resection of the prostate, urethral catheter removal

## Abstract

**Aim:** We aimed to evaluate early versus delayed removal of the indwelling urethral catheter (IUC) following transurethral resection of prostate (TURP). **Methods:** In this clinical trial conducted between July 2016 and June 2020, 90 patients underwent TURP were randomized equally into: group A, early IUC removal (24 h), and group B, delayed IUC removal (72 h). **Results:** The mean length of hospital stay was longer among the patients in group B. There were no significant differences in recatheterization, secondary bleeding, or UTI between groups A and B. The mean VAS score and CRBD were higher in group B. **Conclusion:** Early IUC removal following TURP is safe approach with favorable clinical outcomes.

**Clinical Trial Registration:** NCT04363970 (clinicaltrials.gov)

## Plain language summary

Urethral catheter insertion is an important step after prostate surgery. It may cause urinary infection and distressing symptoms. In this study we evaluated early versus delayed catheter removal, and we found that early IUC removal is safe approach with favorable clinical outcomes.

## Tweetable abstract

For patients undergoing transurethral resection of prostate due to benign prostate hyperplasia, early urethral catheter removal after 24 h is safe approach with favorable clinical outcomes.

## Abstract (plain language summary)

Transurethral resection of the prostate (TURP) remains the gold standard surgical procedure worldwide and is a common, minimally invasive approach in urology practice.

The insertion of an indwelling urethral catheter (IUC) is essential for continuous urinary bladder irrigation to reduce the risk of clot formation and retention.

IUC use is associated with an increased risk of developing urinary tract infection (UTI), prolonged hospital stays, aggravated distressing symptoms.

The timing of IUC removal following TURP is controversial and depends mainly on individual clinical practice.

Early urethral catheter removal after 24 h is safe approach with favorable clinical outcomes.

The clinician can take a decision of early IUC removal following TURP, in order to reduce the hospital stays, cost of treatment and morbidities.

Benign prostate hyperplasia (BPH) is a common urological disease among elderly men and one of the major causes of lower urinary tract symptoms (LUTS). The prevalence of BPH increases with age, as approximately 80% of men older than 70 years suffer from BPH, with a significant negative impact on quality of life [1,2].

Globally, alpha 1 adrenergic receptor antagonists, alone or in combination with 5-alpha-reductase inhibitors, are commonly used as oral medical therapy for LUTS secondary to BPH. Guidelines indicate the need for surgical treatment for patients with severe LUTS or for those suffering from undesirable adverse effects or intolerance to medical therapy [3]. Although several surgical approaches have been developed for the treatment of LUTS secondary to BPH, transurethral resection of the prostate (TURP) remains the gold standard surgical procedure worldwide and is a common, minimally invasive approach in urology practice [4].

Following TURP, the insertion of an indwelling urethral catheter (IUC) is essential for continuous urinary bladder irrigation to reduce the risk of clot formation and retention. However, IUC use is associated with an increased risk of developing urinary tract infection (UTI). Catheter-associated urinary tract infection (UTI) is one of the most common hospital-acquired infections worldwide. Approximately 20% of hospital-acquired bacteremias arise from the urinary tract and are associated with a mortality rate of approximately 10% [^5-7^]. Furthermore, catheter-associated UTIs have been associated with a longer hospital stay, and the economic burden is estimated at $676–12,000 per UTI case and $340–450 million annually [8].

Similarly, prolonged IUC time, UTI and local catheter trauma increase the risk of urethral stricture formation after TURP [9]. Catheter-related bladder discomfort (CRBD) and urethral catheter-related pain (UCRP) are the most common distressing symptoms of TURP [10,11]. CRBD is characterized by symptoms similar to those of overactive bladder, such as urinary frequency and urgency, with or without urge incontinence [12]. Despite diverse medications, many controversies persist in clinical practice and no effective treatment for UCRP and CRBD without adverse events has been established yet [13].

Currently, the optimal time for IUC removal after TURP has not been established. However, it is based on clinical practice rather than evidence-based knowledge, and varies considerably. Moreover, most of the available clinical studies are inconclusive, with variable results [14]. Hence, we conducted this randomized, double blinded, clinical trial to evaluate the effect of early versus delayed IUC removal on the re-catheterization rate, length of hospital stays, secondary bleeding, risk of UTI, UCRP and CRBD.

## Patients & methods

### Study design & approval

This randomized, double blinded clinical trial was conducted between July 2016 and June 2020. The study protocol was approved by the Institutional Review Board at Jordan University Hospital and was registered at ClinicalTrials.gov (NCT04363970). All participants were informed of the study design and signed a written informed consent form in accordance with the Declaration of Helsinki.

### Patient recruitment & randomization

Ninety patients with BPH who underwent TURP were randomized into two equal groups using a computer-generated randomization list. Group A comprised patients in whom the urethral catheter was removed 24 h after the procedure (early removal), and Group B comprised patients in whom the urethral catheter was removed 72 h after the procedure (delayed removal). The randomization order was blinded to the patients, primary surgeons and the post-operative independent observers.

### Inclusion & exclusion criteria

Patients eligible for inclusion in the study were men aged ≥45 years with LUTS secondary to BPH. Patients with large amounts of PVR urine, prostate size more than 100 g, urethral stricture, UTI, simultaneous optical urethrotomy or cystolithotripsy, bleeding diathesis, spinal cord injury, cerebrovascular accident, neurogenic urinary bladder, capsular or urinary bladder perforation or severe bleeding during or after surgery were excluded.

### Technique

All procedures were performed under general or spinal anesthesia by the same experienced surgeon (SA), who was blinded to the randomization order. Intravenous antibiotics were administered to all patients at the time of anesthesia induction and maintained throughout the hospital stay. The patient was then switched to oral antibiotics for 3 days. Monopolar TURP was performed, and the hyperplastic prostate tissue was removed from the surgical capsule. At the end of the procedure three-way urethral catheter 20 Fr was inserted for irrigation with normal saline 0.9%, and the balloon was inflated with 20 cc normal saline. Bladder irrigation was reduced as soon as feasible and stopped if the drainage was clear. Post-operatively, the urethral catheter was removed once the effluent was clear without irrigation, vital signs were stable, and laboratory tests, such as CBC, creatinine and electrolytes, were normal.

### Assessment & outcome measures

All patients were admitted to the hospital and assessed preoperatively by history and physical examination, including International Prostate Symptom Score (IPSS) and digital rectal examination. Laboratory data collected included full blood counts; serum prostate-specific antigen (PSA); kidney function tests, including serum creatinine, urea, sodium and potassium; urine analyses; and urine cultures. Transabdominal ultrasonography was used to assess prostate size and post-void residual urine (PVR). Uroflowmetry tests were performed to measure the maximum flow rate (Qmax).

The outcomes of interest in this study were inability to void re-catheterization, secondary bleeding, UTI, length of hospital stay, CRBD and UCRP. IPSS, Qmax, and PVR were assessed at baseline, 2 weeks, 1 month and 3 months after TURP. UCRP and CRBD were evaluated before and after IUC removal. UCRP was assessed using the VAS score (0–10), with 0 indicating no pain and 10 indicating maximum unbearable pain. CRBD was assessed based on three grades and questionnaire items: grade I (mild 1–3); reported by the patient only on questioning; grade II (moderate 4–6); expressed by the patient without questioning and not accompanied by any behavioral responses; and grade III (severe 7–10); expressed by the patient and accompanied by any behavioral responses. If the VAS score was ≥4, tramadol (1 mg/kg) was administered at the maximum dose of 400 mg/24 h. After urethral catheter removal, all patients were evaluated on the floor by an independent observer before discharge for urinary retention and hematuria. The patients were discharged after they were able to void satisfactorily several times. Adverse events prompting re-catheterization include urinary retention and bleeding. Patients were instructed to visit the emergency room if they developed urinary retention, severe hematuria, or signs and symptoms of urinary tract infection. Patients were assessed at the urology outpatient clinic at different time points during the follow-up for urinary retention, hematuria, UTI, IPSS, PVR and Qmax. At any time point of follow-up, if the patient developed urinary retention and severe hematuria, IUC was reinserted. Patients who developed UTI were treated accordingly. The length of the hospital stay was assessed from the day of admission to the date of discharge.

### Statistical analysis

Descriptive and inferential statistics were calculated using IBM SPSS Statistics for Windows sciences (IBM, 25). According to the level of measurement of the variables, *t*-test and chi-square tests were used to compare the participants' characteristics and clinical outcomes based on the time of catheter removal after TURP. An independent samples *t*-test was used to compare the sample means of the two independent groups for an interval-scale variable when the distribution was approximately normal. The criteria utilized were a power of 80%, a moderate effect size of 0.55, and an alpha of 0.05. The sample size was calculated using G*Power software (Faul *et al.*) [15]. The required sample size for each group is 42. The actual sample size was 90 (45 patients per group).

## Results

**Figure 1.**
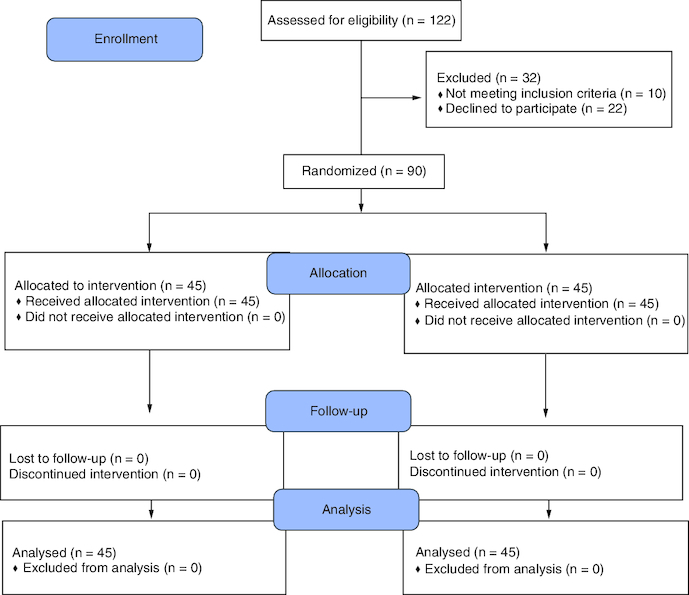
Flow of patients during the study period.

Figure 1 summarizes the patient enrollment, allocation, follow-up, and final analyses in both study groups. A total of 45 patients were included in each group. The mean baseline data and perioperative characteristics were comparable between patients in both groups, with no significant differences. Table 1 presents the baseline and perioperative clinical characteristics of patients.

**Table 1.** Baseline data and perioperative characteristics.

Characteristics	Group A (early removal) (n = 45)	Group B (delay removal) (n = 45)	p-value
Age (years) Mean ± SD	65.84 ± 8.20	68.18 ± 7.99	0.18^†^
BMI (kg/m^2^) Mean ± SD	26.76 ± 3.16	27.63 ± 3.81	0.24^†^
Prostate size (g) Mean ± SD	67.44 ± 16.44	71.22 ± 16.59	0.29^†^
Resection weight (g) Mean ± SD	23.53 ± 7.45	24.13 ± 7.45	0.71^†^
DM (n) (%)	14 (31.1)	20 (44.4)	0.19^‡^
HTN (n) (%)	17 (37.8)	18 (40.0)	0.83^‡^
IHD (n) (%)	11 (24.4)	5 (11.4)	0.11^‡^
α-blockers (n) (%) α-blocker and 5α-reductase (n) (%)	34 (75.6) 11 (4.4)	29 (64.4) 16 (35.6)	0.25^‡^

† *t*-test.

‡ chi-square test.

DM: Diabetes mellitus; HTN: Arterial hypertension; IHD: Ischemic heart disease; SD: Standard deviation.

The mean length of hospital stay was longer among patients in group B than among patients in group A, 3.64 ± 0.57 versus 2.83 ± 0.43, (p < 0.001).

Most patients in both groups voided successfully after IUC removal and had no urinary retention requiring urethral re-catheterization. There were no significant differences in re-catheterization, secondary hemorrhage and UTI between groups A and B 0.07 ± 0.25 versus 0.09 ± 0.29 (p = 0.70), 0.13 ± 0.34 versus 0.09 ± 0.29 (p = 0.51), 0.00 ± 0.0 versus 0.07 ± 0.25 (p = 0.08), respectively. Although there was no significant difference between the groups in terms of UTI, patients in group B who had delayed IUC removal were at a greater risk of developing UTI (p = 0.08) (Table 2).

**Table 2.** Comparison of clinical data.

Parameters	Group A (early removal) (n = 45)	Group B (delay removal) (n = 45)	p-value
Length of hospital stay (days) Mean ± SD	2.83 ± 0.43	3.64 ± 0.57	<0.001^†^
Re-catheterization Mean ± SD	0.07 ± 0.25	0.09 ± 0.29	0.70^†^
Secondary bleeding Mean ± SD	0.13 ± 0.34	0.09 ± 0.29	0.51^†^
UTI Mean ± SD	0.00 ± 0.0	0.07 ± 0.25	0.08^†^
VAS before IUC removal Mean ± SD	2.67 ± 1.04	3.73 ± 1.18	<0.001^†^
VAS after IUC removal Mean ± SD	0.24 ± 0.61	0.56 ± 0.62	0.019^†^
CRBD before IUC removal Mean ± SD	3.36 ± 1.25	5.49 ± 1.20	<0.001^†^
CRBD after IUC removal Mean ± SD	0.22 ± 0.47	1.11 ± 0.57	<0.001^†^
Total Tramadol consumption (mg) Mean ± SD	45.44 ± 59.13	129.0 ± 105.26	<0.001^†^
IPSS at baseline Mean ± SD	24.04 ± 4.41	24.18 ± 4.33	0.62^†^
IPSS at 3 months Mean ± SD	11.92 ± 3.13	12.41 ± 3.73	0.55^†^
Q_max_ at baseline Mean ± SD	8.97 ± 3.59	8.45 ± 3.80	0.51^†^
Qmax at 3 months Mean ± SD	26.78 ± 10.12	25.82 ± 10.06	0.65^†^
PVR at baseline Mean ± SD	96.51 ± 64.26	97.11 ± 60.02	0.96^†^
PVR at 3 months Mean ± SD	27.60 ± 22.47	31.56 ± 22.94	0.41^†^

† *t*-test.

CRBD: Catheter-related bladder discomfort; IPSS: International Prostate Symptom Score; PVR: Post void residual; SD: Standard deviation; UTI: Urinary tract infection; VAS: Visual analog score; Q_max_: Maximum flow rate.

The mean VAS score for UCRP before IUC removal was significantly higher in Group B than in Group A 3.73 ± 1.18 versus 2.67 ± 1.04 (p < 0.001). After the removal of the IUC, the VAS score was still higher among patients in group B 0.56 ± 0.62 versus 0.24 ± 0.61 (p = 0.019). CRBD was significantly higher before and after IUC removal in Group B 5.49 ± 1.20 versus 3.36 ± 1.25 (p < 0.001), 1.11 ± 0.57 versus 0.22 ± 0.47 (p < 0.001), respectively. There were no significant differences between the groups in terms of IPSS, Qmax or PVR at baseline and 3 months after TURP. The mean total dose of tramadol was higher among patients in group B than in group A (129.0 ± 105.26 mg vs 45.44 ± 59.13 mg, respectively); (p < 0.001) (Table 2).

## Discussion

The ideal time for IUC removal after TURP remains controversial, and the decision is usually based on clinical practice rather than on evidence-based knowledge. The major finding of our double blinded, randomized, clinical trial was that early IUC removal after TURP was a feasible and safe clinical approach. Patients in both groups voided successfully, with no significant differences between them in terms of re-catheterization, secondary bleeding and UTI. In addition, the mean length of hospital stay, VAS score and CRBD were lower among the patients in the early IUC removal group.

Yu *et al.* [14] reported in meta-analysis that there was no significant difference in the rate of re-catheterization between the early and delayed catheter removal groups (RR: 1.12, 95% CI: 0.73 1.72). In contrast, different studies have reported that early catheter removal leads to an increased re-catheterization rate and clot retention compared with delayed catheter removal [^16-18^]. In our study, the mean re-catheterization rate was 0.07 ± 0.25, (p = 0.70).

Chander *et al.* [19] demonstrated in their study that earlier catheter removal reduced the length of hospital stay from 3.1 to 1.28 days. Similarly, Shum *et al.* [20] concluded that early catheter removal on the first post-operative day was safe, with an overall hospital stay of 1.6 days. However, this study had a small sample size (40 patients) and the energy source for TURP was bipolar, whereas we used mono-polar. Our results confirm the previous finding that the length of hospital stay was significantly reduced in the early catheter removal group to 2.83 ± 0.43 versus 3.64 ± 0.57 (p < 0.001).

Furthermore, several studies have reported that early catheter removal is not only safe but also cost-effective. Mueller *et al.* [21] reported that the mean cost savings for early catheter removal following TURP were $829 and $1406 for patients aged <70 and >70 years, respectively.

Secondary bleeding is mainly attributed to surgical techniques and patient factors such as bleeding diathesis, large prostate volume and comorbidity [22]. One variable that can predispose patients to perioperative bleeding is UTI [23]. We ensured that all patients had UTI preoperatively and were treated preoperatively. We also administered prophylactic antibiotics at the time of induction and continued the procedure post-operatively. In addition, Chander *et al.* [19] did not find significant bleeding or clot retention after early catheter removal. They reported early catheter removal within 7.5 h in 92% of patients and within 10 h in the remaining 8% of their patients. None of the patients required re-catheterization due to bleeding or clot retention. Yu *et al.* [14] reported in meta-analysis that there were no significant differences in the rate of secondary hemorrhage between the early and delayed catheter removal groups (RR: 1.07, 95% CI: 0.54 2.13).

IUC is a common cause of UTI, as it increases the risk of infection by 5–10% per day of use [24]. The expert panel of the Infectious Diseases Society of America agreed with evidence-based international clinical practice guidelines for procedures and strategies to reduce the risk of catheter-associated asymptomatic bacteriuria and UTI. They concluded that there is strong evidence that the IUC should be removed as soon as it is no longer required to minimize the risk of bacteriuria and UTI [7]. In the present study, the mean UTI in both groups was comparable, but patients in group B who underwent late IUC removal were more likely to have UTI (p = 0.08).

UCRP and CRBD can trigger serious behavioral effects, such as confusion and agitation, which can lead to traumatic attempts to remove the urethral catheter, causing urethral injury and subsequent urethral stricture [10,^25-27^]. Muscarinic receptor antagonists (MRA) have been shown to be effective in improving tolerance IUC's [10,25]. However, this approach has proven disadvantages related to undesirable adverse effects and pain, which seem to be unrelated to the muscarinic receptors. In our study, the mean VAS and CRBD scores before and after IUC removal were lower in group A, and this is expected because the duration of IUC in group A was shorter (24 h vs 72 h).

This study has some limitations. First, the sample size is small. Second, we did not provide a validated assessment of the cost and quality of life. Finally, there is no validated questionnaire to measure CRBD. Despite these limitations, this study was a randomized controlled trial and all patients, primary surgeons and post-operative independent observers, were blinded to the randomization at the end of the surgery and post-operative follow-up.

## Conclusion

Our data indicate that early IUC removal following TURP is a safe and feasible clinical approach that does not increase the incidence of recatheterization, secondary bleeding and UTI. Furthermore, the length of hospital stays, UCRP levels, CRBD and analgesic consumption were significantly reduced. Based on our clinical results, the clinician can take a decision of early IUC removal following TURP, in order to reduce the hospital stay, cost of treatment and morbidities.

## Data sharing statement

The authors certify that this manuscript reports original clinical trial data (clinical trial registration number NCT04363970). Individual, de-identified participant data and study protocols will be available beginning 9 months and ending 36 months following article publication. The use of this shared data is in accordance with the following terms agreed upon their receipt: investigators whose proposed use of the data has been approve and an independent review committee identified for this purpose and for individual participant dale-meld analysis.
